# Continuity of care and integration between hospital and primary care: the experience of the Local Health Authority of Ravenna

**Published:** 2012-09-04

**Authors:** Tiziano Carradori

**Affiliations:** Segreteria Direzione generale, Ausl di Ravenna, Italy

The present epidemiologic context is defined by the progressive ageing of the population and the increasing of chronic conditions.

It is necessary to diversify the health care answer and the coordination continuity of care that was confirmed from the literature. A new figure of “medico di riferimento” (MDR) plays the role to manage the care pathway and to respond to the exigency of putting the person in the centre of the care pathway and obtain a better integration of cares.

Powerpoint presentation available at http://www.integratedcare.org at congresses – San Marino – programme.

